# COVID-19 Outbreak Associated with Air Conditioning in Restaurant, Guangzhou, China, 2020

**DOI:** 10.3201/eid2611.203774

**Published:** 2020-11

**Authors:** Jianyun Lu, Zhicong Yang

**Affiliations:** Guangzhou Center for Disease Control and Prevention, Guangzhou, China

**Keywords:** 2019 novel coronavirus disease, COVID-19, severe acute respiratory syndrome coronavirus 2, SARS-CoV-2, viruses, respiratory infections, zoonoses, rebuttal, family cluster, restaurant, droplet transmission, outbreak, China

**In Response:** We thank Prof. Rule (*1*) for her comments on our letter (*2*). We welcome the opportunity to offer additional information on several of the points made.

**Figure 1:**
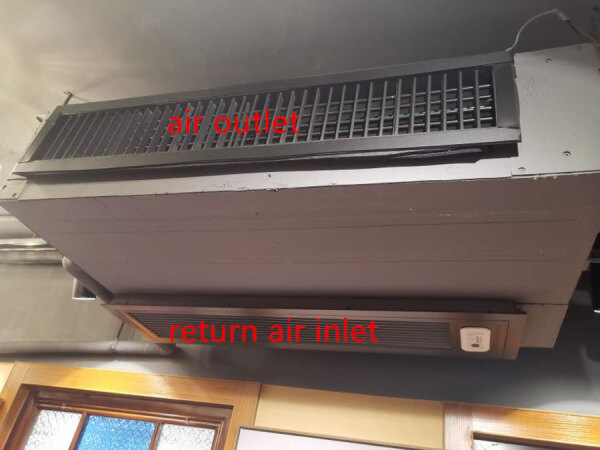
Inlet and outlet of air conditioner described in study of COVID-19 outbreak associated with air conditioning in restaurant, Guangzhou, China, 2020 (*2*).

We wish to explain that although she stated that “‘The air outlet and the return air inlet for the central air conditioner were located above table C (Figure 1, panel B)’ does not describe the actual layout depicted in the figure, in which the air conditioner is located by table C and the exhaust fan is between tables B and D” (*1*). In fact, the air outlet and the return air inlet for the central air conditioner were located above table C (Figure 1). The central air conditioner is constructed in 2 parts: air outlet and air inlet, indicating no discrepancy between the text and the figure.

We agree that virus transmission in this outbreak could be explained by droplet transmission and the possibility that persons move around, touch surfaces, go to the restroom, or engage in other close contact. We considered these scenarios but omitted their mention in the research letter. Instead, we reported what we considered to be the most likely scenario, which is that the droplet transmission was prompted by the air blown from right to left and then across tables C, A, and B successively. The air movement facilitated dispersion of droplets containing severe acute respiratory syndrome coronavirus 2 (SARS-COV-2) from patient A1 to the 3 patients at table B. After the air had passed over tables C, A, and B, patients C1 and C2 acquired the infection from droplets mixed with SARS-COV-2 as they returned to the air inlet over table C. None of the 62 persons at the other 12 tables were infected, which suggests that the alternate scenarios (touching surfaces or going to the restroom at the same time) were less likely. Furthermore, some diners and waitresses also went to the restroom and were not infected. In addition, closed-circuit television tapes did not show that the patients in our study had gone to the restroom at the same time. The tapes showed that patient C1 walked in and out several times and passed table A on the way, which might be one of the reasons for the infection of patient C1.

**Figure 2:**
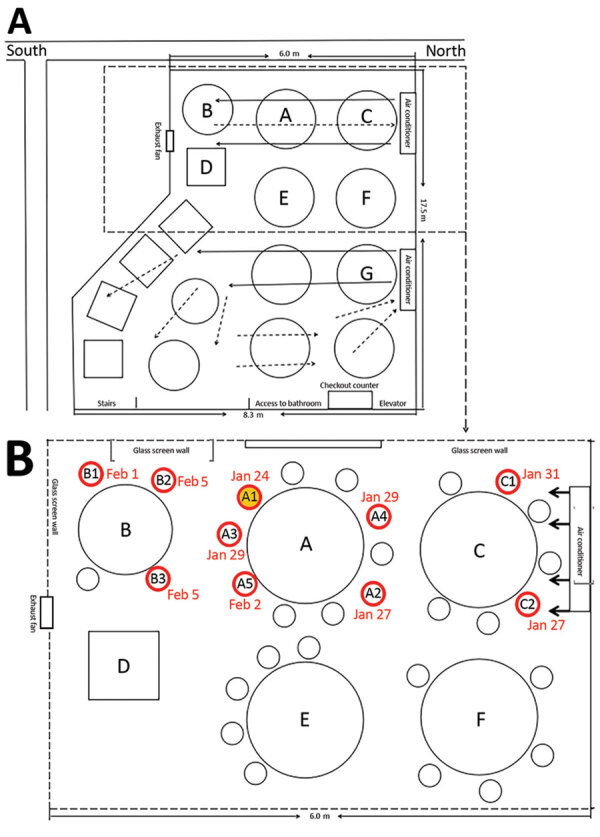
Air flow (A) and seating diagram (B) for restaurant described in study of COVID-19 outbreak associated with air conditioning in restaurant, Guangzhou, China, 2020 (*2*).

We did not describe the exhaust fan in the text, but we drew an exhaust fan between tables B and D in figure. To point out the exhaust fan, we drew it bigger than actual measurements, which were only 12 × 12 inches (305 mm × 305 mm); the fan was not strong enough to remove all air produced by the central air conditioner. After the initial publication, we revised the figure to show the appropriate size of the exhaust fan and added details about its size, emphasizing that the ventilation system was not well designed (Figure 2). The main air flow is discharged from the central air conditioning outlet and then returned to the air inlet. Because of the weak exhaust system, the ventilation in the restaurant was not good. We did not ignore the presence of the exhaust fan.

We conclude that the air conditioner prompted transmission of SARS-CoV-2; the customers in the airflow were at high risk for infection with SARS-CoV-2 in the poorly ventilated environment. Because the staff and other diners were not exposed to the airflow mixed with SARS-CoV-2 transmitted by patient A1, their risk for infection was lower.

We excluded the possibility of aerosol transmission (*2*). It has been reported that aerosols (<5 μm) can remain in the air and disperse long distances (>1 m) (*3*). The potential for aerosol transmission has been reported for severe acute respiratory syndrome coronavirus (*4*) and Middle East respiratory syndrome coronavirus (*5*,*6*). However, in our study, none of the 62 persons at the other 12 tables were infected. Moreover, the smear samples from the air conditioner were all negative by reverse transcription PCR.

We believe that the most likely scenario for SARS-CoV-2 transmission in the restaurant was droplet transmission prompted by the air conditioning, although other scenarios are possible. The exhaust fan was not strong enough to modify the ventilation; the main mode of air circulation was the central air conditioning outlet with air returned to the air conditioning inlet. Because of the weak exhaust system, ventilation in the restaurant was poor. We recommend that air handling systems be sufficiently powered and maintained.
